# Personalized Flexible Meal Planning for Individuals With Diet-Related Health Concerns: System Design and Feasibility Validation Study

**DOI:** 10.2196/46434

**Published:** 2023-08-03

**Authors:** Maryam Amiri, Juan Li, Wordh Hasan

**Affiliations:** 1 Department of Computer Science North Dakota State University Fargo, ND United States

**Keywords:** diabetes, fuzzy logic, meal planning, multicriteria decision-making, optimization

## Abstract

**Background:**

Chronic diseases such as heart disease, stroke, diabetes, and hypertension are major global health challenges. Healthy eating can help people with chronic diseases manage their condition and prevent complications. However, making healthy meal plans is not easy, as it requires the consideration of various factors such as health concerns, nutritional requirements, tastes, economic status, and time limits. Therefore, there is a need for effective, affordable, and personalized meal planning that can assist people in choosing food that suits their individual needs and preferences.

**Objective:**

This study aimed to design an artificial intelligence (AI)–powered meal planner that can generate personalized healthy meal plans based on the user’s specific health conditions, personal preferences, and status.

**Methods:**

We proposed a system that integrates semantic reasoning, fuzzy logic, heuristic search, and multicriteria analysis to produce flexible, optimized meal plans based on the user’s health concerns, nutrition needs, as well as food restrictions or constraints, along with other personal preferences. Specifically, we constructed an ontology-based knowledge base to model knowledge about food and nutrition. We defined semantic rules to represent dietary guidelines for different health concerns and built a fuzzy membership of food nutrition based on the experience of experts to handle vague and uncertain nutritional data. We applied a semantic rule-based filtering mechanism to filter out food that violate mandatory health guidelines and constraints, such as allergies and religion. We designed a novel, heuristic search method that identifies the best meals among several candidates and evaluates them based on their fuzzy nutritional score. To select nutritious meals that also satisfy the user’s other preferences, we proposed a multicriteria decision-making approach.

**Results:**

We implemented a mobile app prototype system and evaluated its effectiveness through a use case study and user study. The results showed that the system generated healthy and personalized meal plans that considered the user’s health concerns, optimized nutrition values, respected dietary restrictions and constraints, and met the user’s preferences. The users were generally satisfied with the system and its features.

**Conclusions:**

We designed an AI-powered meal planner that helps people create healthy and personalized meal plans based on their health conditions, preferences, and status. Our system uses multiple techniques to create optimized meal plans that consider multiple factors that affect food choice. Our evaluation tests confirmed the usability and feasibility of the proposed system. However, some limitations such as the lack of dynamic and real-time updates should be addressed in future studies. This study contributes to the development of AI-powered personalized meal planning systems that can support people’s health and nutrition goals.

## Introduction

### Background

Chronic diseases such as diabetes and heart disease are major health challenges that affect millions of people worldwide. One of the key strategies for managing chronic diseases is healthy eating, which can help prevent, delay, or improve the symptoms and complications associated with these conditions [1]. Meal planning is a useful tool for achieving healthy eating goals, as it can help individuals with chronic diseases ensure they are consuming a balanced diet that provides the necessary nutrients for good health [2]. For instance, meal planning can help regulate blood glucose levels in individuals with diabetes by balancing the intake of carbohydrates, proteins, and fats [3]. Meal planning can also help lower the risk of high blood pressure and high cholesterol in individuals with heart disease by promoting a diet that is low in saturated fat and high in fruits, vegetables, and whole grains [4]. Furthermore, meal planning can help individuals with chronic diseases manage their weight and adhere to their medication schedule by avoiding overeating and planning meals around their treatment regimen [5]. Therefore, meal planning can play a crucial role in managing chronic diseases and improving the overall health outcomes.

With the overwhelming number of food and nutrition websites and amount of information out there [6-9], it is difficult for a busy person to decide exactly what is appropriate for them. Keeping track of all health guidelines and their limitations is difficult for many people. Furthermore, many other personal conditions need to be considered, such as the time limit for preparing meals, budget constraints, styles of cuisine, religious requirements, cultural factors, and traditions. Besides providing healthy and nourishing meal options, planning meals can help people save time during meal preparation. This can eliminate the stress of figuring out meals every day. Recently, there have been various mobile apps (eg, EatRight, MyPlate, FDA, MyFitnessPal, Lifesum, CalorieCounter, and Fooducate [10-17]) that help people to plan their meals. These apps tend to provide generic planning and recommendations without considering the user’s special health conditions and other personal preferences or considering only a limited fixed factor [18-20]. These limitations include the limited consideration of personalized health constraints, lack of customization for taste preferences, and incomplete consideration of cultural and regional dietary practices.

### Objective

There are no one-size-fits-all diets. To help people with health concerns make healthy and sustainable meal plans, we proposed an artificial intelligence (AI)–enabled personalized meal planning strategy tailored to an individual’s unique dietary needs, preferences, and goals while also considering their lifestyle and available resources. Specifically, we adopted semantic web technologies to model complex and heterogeneous diet knowledge, used semantic logic to enable automatic machine reasoning to apply clinical diet rules, used fuzzy logic to handle uncertainty and vagueness of food data and improve flexibility, and designed heuristic search–assisted multicriteria decision-making (MCDM) to effectively integrate multiple user preferences in meal planning. In our work, we treated each person as a unique individual with different health concerns, flavor preferences, financial situations, religions, and cultural and traditional backgrounds.

Our app addresses the gaps in existing meal planning systems by offering a more comprehensive and personalized meal planning experience that considers a wide range of factors, including health constraints, taste preferences, cultural practices, changing goals, and emotional connections to food. In addition, our approach used highly flexible and efficient AI algorithms, enabling us to consider a significantly larger number of factors and deliver fast and flexible responses. This further distinguishes our app from existing solutions as it allows for more comprehensive and dynamic meal planning capabilities. We implemented the proposed planning system with a mobile app prototype. Comprehensive evaluation tests were performed on the prototype.

## Methods

### System Overview

**Figure 1:**
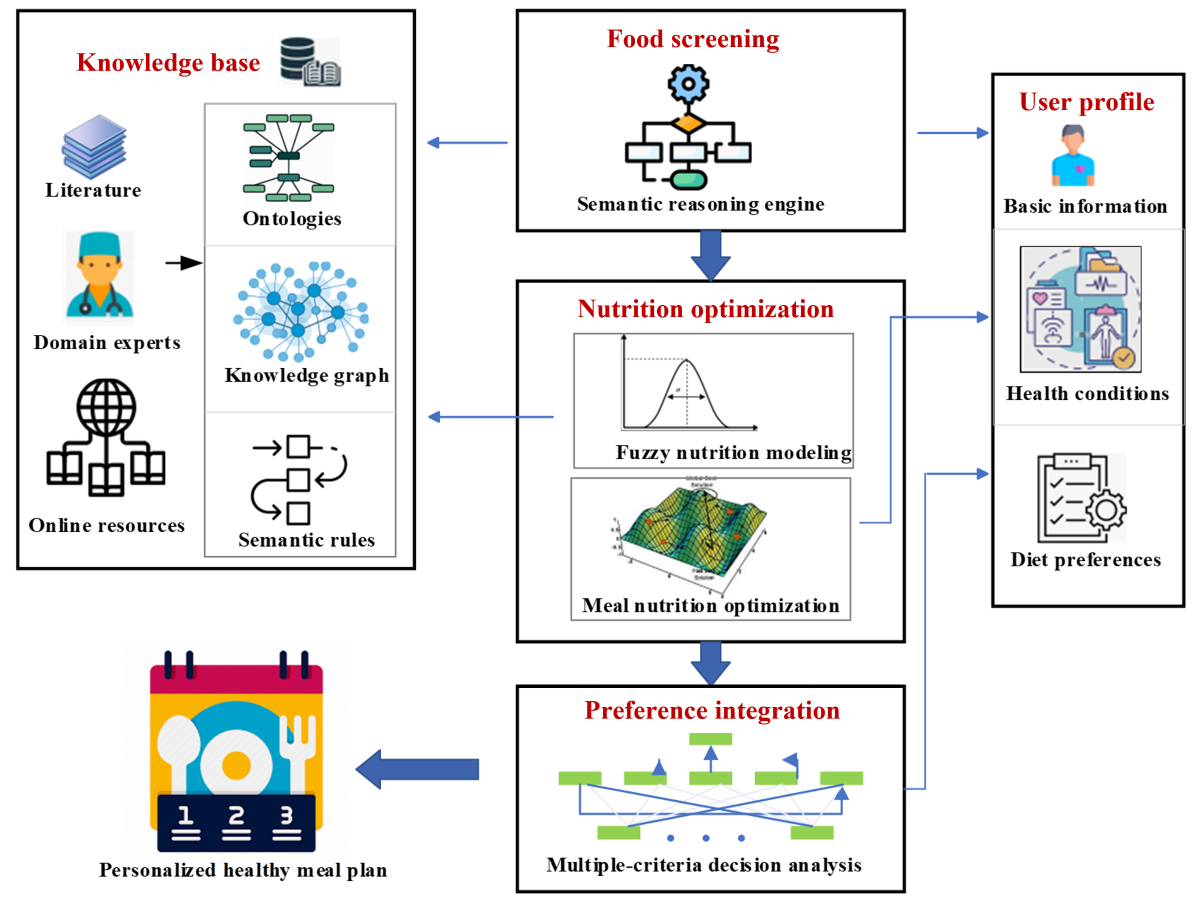
Proposed planning system architecture.

Figure 1 shows the architecture of the proposed planning system. The system’s brain is a comprehensive knowledge base that includes knowledge about food, nutrition, and clinical guidelines for healthy eating regarding different health concerns. The meal planning system depends on this knowledge to understand food nutrition values and healthy meal requirements. The system also maintains a user profile, including the user’s basic physical and economic information, health concerns, and diet constraints and preferences. The user profile is the foundation for the personalization of the meal planning system. On the basis of the user’s health conditions, corresponding healthy eating guidelines, represented as semantic rules, can be applied to screen food and meals so that they can satisfy healthy eating guidelines. A fuzzy membership scheme was used to model the desirability of nutrition intake, providing more flexibility and tolerance to the vagueness of data. We designed an optimization function to identify meals with optimal nutrition value. To integrate users’ other preferences, we proposed a novel, multicriteria decision analysis mechanism assisted by a heuristic search method that can efficiently locate meals that satisfy multiple, conflicting user preferences. In the following sections, we’ve presented each of the system components.

### Knowledge Foundation

The brain of the planner is a comprehensive food and nutrition knowledge graph [21]. It is a visual representation of information and the relationships between various elements of food and nutrition. This includes information on food groups, nutrients, dietary recommendations, and the relationship between food consumption and health outcomes. A knowledge graph can help provide a comprehensive and interconnected view of food and nutrition, making it easier to understand and access the information. The graph data were collated from a variety of reliable sources, such as the US Department of Agriculture’s food and nutrition data set [22] and FoodKG [23]. Some of these sources were structured data sets and some others were unstructured and needed to be transformed into structured graph knowledge. By leveraging these diverse sources, we aimed to cross-validate the information and mitigate the risk of relying solely on a single data repository. Knowledge graphs enable the fast and easy navigation of data and support automatic reasoning and inferring new knowledge. The knowledge graph can be extended to national or international dietary guidelines, such as the US Department of Agriculture’s Dietary Guidelines for Americans or the World Health Organization’s Global Recommendations on Physical Activity for Health. It also includes diet guidelines for different kinds of health concerns; for example, to get food and nutrition guidelines for diabetes, we adopted the guidelines from multiple resources, such as the American Diabetes Association, the British Dietetic Association, the Association of Clinical Endocrinologists, and the American College of Endocrinology.

**Figure 2:**
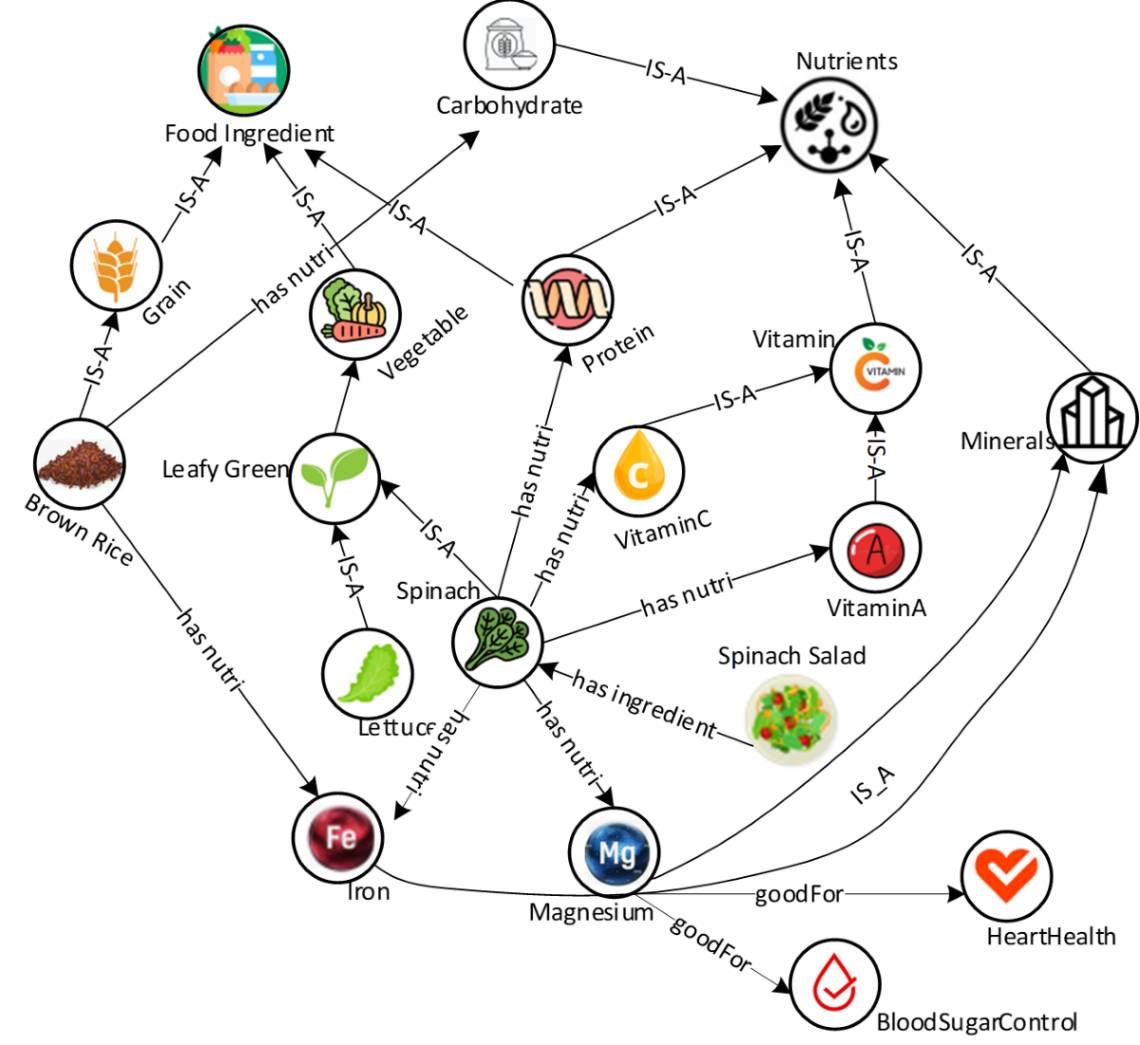
Part of the food and nutrition knowledge graph. “IS-A” denotes subclass relationship, indicating 1 class is a subclass of another. “has-nutri” represents “has-nutrition”.

Figure 2 shows part of our food and nutrition knowledge graph. Nodes represent entities, such as foods, nutrients, dietary recommendations, and health outcomes, whereas edges represent the relationships between these entities. Food items may be linked to the nutrients it contains, whereas a nutrient node may be linked to the recommended daily intake and the health outcomes associated with inadequate or excessive intake. For example, from the knowledge graph, we can see that spinach is a leafy green vegetable. It has the nutrient magnesium, which is beneficial for heart health and blood sugar control.

In addition to food and nutrition knowledge, a comprehensive user profile includes users’ biological, socioeconomic, and cultural characteristics and contextual situations that influence peoples’ food choices. We used a biocultural user profile ontology [10] to model the user profile.

### Rule-Based Screening

The first step in meal planning is to screen food ingredients that violate the user’s mandatory constraints, such as medical, allergy, cultural, and religious constraints. For example, if a user is allergic to peanuts, peanuts as an ingredient should be eliminated from meal ingredient lists. Alternatively, if a user is a vegetarian, animal products as ingredients must be eliminated. Subsequently, rule-based food screening uses a set of predefined rules to evaluate the nutritional value of food choices and to make recommendations. For example, rules will be applied to evaluate a food based on its calorie content, fat content, and the presence of certain vitamins and minerals, and then a recommendation will be made based on those evaluations. The system may flag foods that are high in calories, unhealthy fats, or lack certain essential nutrients, and suggest healthier alternatives. In addition to food ingredients, rules can be applied to screen meals. For example, a user with type 2 diabetes should have 3 to 5 carbohydrate choices (each choice has 15 g) for every meal, based on the calculated estimated energy requirements. A user with hypertension should not consume more than 2300 mg and not less than 500 mg of sodium per day, with an ideal limit of 1500 mg. In addition, the cholesterol intake of users with hypertension should be restricted to less than 300 mg per day.

We used semantic rules, which are description logic in nature, to apply these dietary recommendations. For example, for the rule about sodium intake: *Sodium should be less than 1500 mg per day for people with diabetes and high blood pressure* [24], the Semantic Web Rule Language rule is defined as follows:

Person(?user) ^hasHealthConcern(?user,Hypertension) ^hasMeal(?user,?meal) ^hasSodium(?meal,?sodium) ^swrlb:greaterThan(?sodium, 1500) -> isRecommended(?meal,false)

We implemented a reasoner that uses forward chaining [25] as the implementation strategy, which can be described logically as repeated applications of modus ponens [26]. The inference engine uses forward chaining searches of the inference rules until it finds the one where the antecedent is known to be true. When such a rule is found, the engine can conclude or infer the consequence, resulting in the addition of new information to its data.

### Fuzzy-Based Nutrition Optimization

#### Overview

Incorporating fuzzy membership into our planning system allows for more informed decisions regarding food choices and nutrient intake, considering the uncertainties and subjectivity inherent in food preferences, dietary restrictions, and health goals. Fuzzy logic, using linguistic variables such as “low,” “medium,” and “high” provides flexibility compared with strict binary decision rules, effectively capturing uncertainty and improving recommendation accuracy and personalization. The application of fuzzy membership enhances the accuracy, personalization, and flexibility of food and nutrition recommendations.

#### Fuzzy Interpretation of Dietary Reference Intakes

Fuzzy sets represent vague information without clear boundaries, in contrast to crisp sets that classify objects as belonging or not belonging. Although most current dietary rules and guidelines use crisp sets based on dietary reference intake, we encounter limitations. For instance, following the dietary approaches to stop hypertension diet recommendation, the limit for sodium intake is within 2300 mg per day [24]. A sodium intake of 2305 mg would be deemed completely unacceptable under crisp logic. However, fuzzy membership allows for a more nuanced approach, defining the degree of desirability for the recommended amounts. We used fuzzy membership functions to estimate nutrient intake between 0 (not desired) and 1 (completely within the desired range), overcoming the limitations of crisp logic decisions. Curve fitting based on key points, such as extreme and optimal intake levels, was used to construct the membership function. Details of the curve fitting algorithm are illustrated in Figure S1 in Multimedia Appendix 1 [27-29], which illustrates the fuzzy membership functions for various nutrients, such as protein, fat, fiber, sugar, sodium, and carbohydrate.

#### Nutrition Optimization

To produce the optimal intake of nutrients in a meal, each nutrient has a fuzzy set in which the membership value should achieve its maximum value (µ=1). We adopted the Prerow Value (PV) proposed by Wirsam et al [27] to measure the closeness of a meal’s nutrients to the optimal recommended value. PV is the product of the minimal membership value and harmonic mean of the fuzzy sets of the remaining observed nutrients, as defined in equation 1. PV is graded between 0 and 1, and nutrition with the lowest value has the most influence on the result.



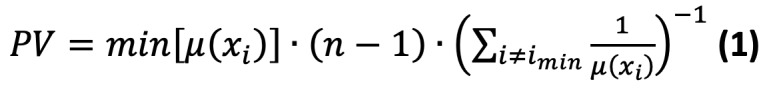



On the basis of the research by Wirsam [27], the preferred PV values were >0.7 and the optimal PV values were >0.9.

To determine the best combination of meals for a day (breakfast, lunch, and dinner), we proposed a heuristic optimization algorithm that computes the optimal PV value. In this algorithm, we generate a population of unique meals, denoted as *x*. Each one of these *x* meal is considered as a potential candidate for an optimal daily meal plan encompassing 3 meals: breakfast, lunch, and dinner. As the iterations progress, each meal in the *x* population will be separately enhanced, allowing the daily menus to develop independently. The PV is used as the fitness value to evaluate all meals. Global-best is a list sorted by size y that saves y-best meals based on the fitness value (y<x). The meal that has the lowest PV is selected to be replaced with a better meal that has a better amount of nutrition. This replacement is based on the summation of the nutrition in all the foods in a day toward the optimal fuzzy membership value. The details of the fuzzy nutrition optimization algorithm are presented in Figure S2 in Multimedia Appendix 1.

### Multiobjective Optimization

Besides nutrition, other factors may affect a user’s satisfaction with a meal, such as the cost, preferred cuisine flavor, color, texture, and temperature, cooking time, and rating of the recipe on the website. Many of these factors may conflict with one another. We proposed an MCDM approach to determine the best daily meals that a user likes. The basic idea of the algorithm is to use the combined Technique for Order of Preference by Similarity to Ideal Solution (TOPSIS) [30] and analytic hierarchy process [31] methods to choose the best meals based on multiple criteria.

The algorithm mainly includes defining the criteria for choosing the best meal, determining the weighting of each criterion based on the user’s preferences using analytic hierarchy process, normalizing the criteria values, calculating the weighted normalization of each meal, determining the positive and negative ideal solutions, calculating the Euclidean distances, determining the relative closeness of each meal to the positive ideal solution, and finally sorting the meals based on the relative closeness values to find the best meal. The algorithm provides a systematic way to consider user preferences and make an informed decision about which meal is the best option. The details of the algorithm can be found in Figure S3 in Multimedia Appendix 1.

### Ethics Approval

This study underwent an ethics review and received the necessary approval from the North Dakota State University Institutional Review Board (IRB0003985).

### Informed Consent

Informed consent was obtained from all the participants through an email-based consent process. The participants were provided with detailed information about the study and its objectives, procedures, and potential risks and benefits. The consent email clearly stated that participation in the study was voluntary, and the participants were informed that they could withdraw their participation at any time without any consequences. By following the provided link and participating in the study, participants indicated their voluntary consent to participate in the research.

### Privacy and Confidentiality Protection

Questions were taken to protect the privacy and confidentiality of human subjects. The study data were anonymized to ensure participant anonymity.

## Results

### System

To evaluate the proposed meal planning system, we implemented a mobile app as a proof-of-concept prototype system. We collected recipes from multiple well-known recipe websites, such as the New York Times, Food [32], and Epicurious, as candidate meal options. A crawler was built to collect recipes. Preprocessing was performed on these recipes, including removing incomplete recipes and merging highly similar recipes. A recipe parser was developed to extract key information, such as ingredients, amount, unit, and cooking time, from the recipes. This information was mapped using a predefined food nutrition ontology. The extracted information is stored in a structured recipe data set. The data set contains 176,206 meal recipes and 563 food tags.

User information, such as sex, age, health issues, and diet preferences, was collected through a short survey in the app. Furthermore, users had the flexibility to update or modify these settings at any time through the “Settings” menu within the app. This allows users to adjust their information as needed, ensuring that meal plans align with their current preferences and requirements.

**Figure 3:**
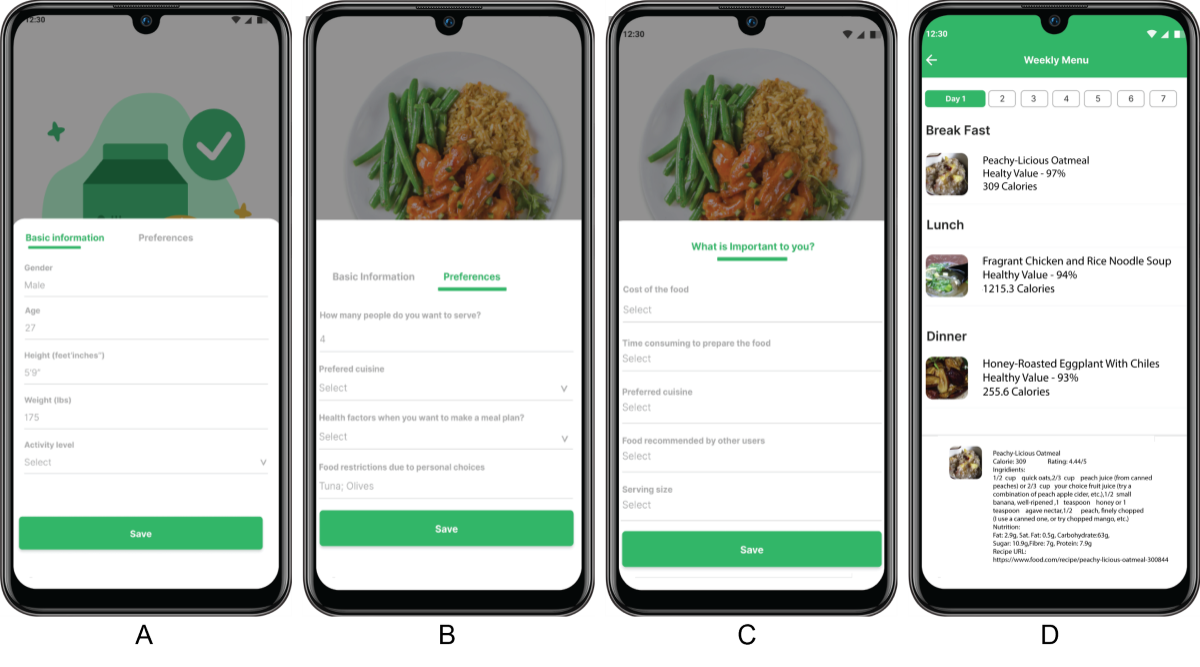
App screenshots: (A) the collection of basic information, (B) and (C) survey of user's diet preferences, and (D) the app providing recommended meals.

Each time, meal planning can make 1 week’s meal plan for the user. Figure 3 shows screenshots of the app. Figure 3A shows the user’s basic information that is collected. Figure 3B and 3C show the user’s diet preferences surveyed. Figure 3D shows the app providing recommended meals to the user. In Figure 3D, the “Healthy Value” represents the PV value defined previously. To enhance user-friendliness, we used an easily understandable name instead of directly referencing the PV value. The current version of the app was designed to generate meal plans based on a conventional 3-meal structure. Future iterations of the app may include options to adjust the number of meals during the day or incorporate snack options.

### Use Case Study

To evaluate the system’s usability from the user’s point of view, we created use cases before providing the system to real users. We listed 2 use cases that represent different scenarios and preferences. User 1, a 45-year-old female user, has hypertension and is allergic to peanuts. Her BMI value is 25.8 kg/m^2^. She cares about the cost of the meals and prefers more economical meals. She lives with 3 other family members, so she cares about the number of servings. The time required to prepare food is less important to consider. User 1 likes Mexican and vegetarian cuisines, but she is open to trying new tastes. She always looks at the rating number of meals (a score given by other users who have tried the meal and rated it on a scale of 1-5 stars) to choose meals. User 2, a 30-year-old male user, has type 2 diabetes. His BMI value is 26.9 kg/m^2^. It is important to consider the time needed to prepare food as he is busy. User 2 prefers food from his favorite cuisine list and likes Asian cuisines. He sometimes looks at the rating number of meals to choose meals. The cost of meals is not very important to him.

Table 1 shows an example of a daily meal for both users. The recommended meal plans were optimized based on 176,206 meal options in our database, considering users’ preferences and specific health constraints. All the recommended meals were verified by human experts that the meals satisfy the users’ health guidelines or constraints and follow users’ preferences. Each meal also has a health value percentage (a score calculated by our app based on how well the meal meets the user’s nutritional and health needs) that helps users compare and select meals.

**Table 1:** Recommended meal^a^.

	User 1: hypertension (PV^b^=0.98549)				User 2: diabetes (PV=0.92071)		
	Title	Nutrition	Information	Title		Nutrition	Information
Breakfast	Veggie Omelet	Calories: 560.1, carbohydrate: 42.8 g, sodium: 495 mg, sugar: 6.1 g, fiber: 9.6 g, protein: 47.2 g, fat: 37.3 g, saturated fat: 3.7 g, cholesterol: 7 mg	Time: 20 min, serves: 4, rating: 5, reviews: 2, flavor: 1, cost: US $6	Couscous for Breakfast!		Calories: 720, carbohydrate: 48.5 g, sodium: 159 mg, sugar: 0.4 g, fiber: 4.2 g, protein: 12.8 g, fat: 10.5 g, saturated fat: 3.9 g, cholesterol: 21.4 mg	Time: 6 min, serves: 2, rating: 5, reviews: 2, flavor: 1, cost: US $8
Lunch	Black Bean Spaghetti	Calories: 1150, carbohydrate: 55.8 g, sodium: 458 mg, sugar: 7.5 g, fiber: 14 g, protein: 26.1 g, fat: 12.3 g, saturated fat: 4.1 g, cholesterol: 14.7 mg	Time: 40 min, serves: 4, rating: 5, reviews: 3, flavor: 0, cost: US $10	Dum Ka Chicken		Calories: 780.3, carbohydrate: 18.05 g, sodium: 443.6 mg, sugar: 10.2 g, fiber: 4.5 g, protein: 75.7 g, fat: 42.7 g, saturated fat: 13 g, cholesterol: 236.2 mg	Time: 55 min, serves: 2, rating: 4, reviews: 2, flavor: 1, cost: US $17
Dinner	Mexican Macaroni and Cheese	Calories: 461.4, carbohydrate: 55.8 g, sodium: 182.3 mg, sugar: 11.1 g, fiber: 4.8 g, protein: 31.6 g, fat: 12.5 g, saturated fat: 3.7 g, cholesterol: 96.9 mg	Time: 45 min, Serves: 2, rating: 5, reviews: 2, flavor: 1, cost: US $8	Curried Winter Vegetable Soup		Calories: 685.6, carbohydrate: 66.3 g, sodium: 713.6 mg, sugar: 18.2 g, fiber: 23.2 g, protein: 16.7 g, fat: 13.1 g, saturated fat: 1.8 g, cholesterol: 0 mg	Time: 40 min, serves: 4, rating: 5, reviews: 4, flavor: 0, cost: US $10

^a^Total nutrition of the day: user 1—fat: 62.1 g, sodium: 1135.3 mg, sugar: 24.7 g, fiber: 28.4 g, protein: 104.9 g, and carbohydrate: 171.2 g. User 2—fat: 66.3 g, sodium: 1316.2 mg, sugar: 28.8 g, fiber: 31.9 g, and protein: 105.2 g.

^b^PV: Prerow Value.

To ensure the accuracy and reliability of the nutritional information and health constraints provided by the app, we conducted manual verification of a randomly selected sample of meals produced from various use cases. We are pleased to report that all the verified samples were found to be 100% accurate in terms of their nutritional information and adherence to health constraints.

### User Study

We conducted a user study to evaluate the usability and performance of our mobile app in meal planning.

#### Compensation

No compensation was provided to participants involved in the study. The study was conducted on a voluntary basis, and the participants did not receive any form of monetary or nonmonetary compensation.

#### Design

To recruit a diverse and representative sample of potential users for our app, we used web-based platforms and flyers to advertise the study. Interested participants were invited to complete a screening questionnaire. On the basis of the screening results, we selected 39 adults who met our inclusion criteria and consented to participate in the study. By selecting a sample size of 39 participants, we were able to strike a balance between having a reasonable number of participants to obtain meaningful insights while still being feasible within the constraints of our study. The demographic information of the participants is provided in Table 2, with a higher proportion of female participants, possibly reflecting sex differences in meal preparation behavior. Among the participants, 1 had diabetes, 3 had hypertension, and the remaining participants had no health concerns.

During the study, users were asked to provide individual-level information that is relevant for personalizing their meal plans. This information included age, which helped determine the appropriate caloric intake and nutrient requirements for different life stages. Sex was considered to account for physiological differences and specific needs between male individuals and female individuals. Data on medical conditions were also collected to identify any chronic diseases or dietary restrictions that could impact the suitability of certain foods or nutrients.

In addition, users were prompted to select their physical activity level based on the World Health Organization’s guidelines and recommendations [33-36]. This information indicated the amount of physical activity that users typically engaged in per week. It allowed us to adjust caloric intake and nutrient requirements based on their energy expenditure and physical activity goals. We defined 4 physical activity levels: sedentary (<150 min of moderate-intensity or 75 min of vigorous-intensity physical activity/wk), low active (150 min of moderate-intensity or 75 min of vigorous-intensity physical activity/wk), active (>150 min but <300 min of moderate-intensity or >75 min but <150 min of vigorous-intensity physical activity/wk), and very active (≥300 min of moderate-intensity or ≥150 min of vigorous-intensity physical activity/wk).

The participants were introduced to the app through a detailed description of the user interface, features, and functionalities. They were given demonstrations and explanations of how the app worked, enabling them to gain a clear understanding of its capabilities. Furthermore, the participants received training sessions before using the app. These sessions focused on familiarizing them with the app’s interface, navigation, and various functionalities. The training aimed to ensure that participants felt comfortable and confident in using the app and in understanding its features.

**Table 2:** Demographic information of test participants (n=39).

Variables			Participants, n (%)
**Age group (years)**			
	18-24	7 (9)	
	25-34	17 (23)	
	35-44	8 (11)	
	45-54	5 (7)	
	55-64	2 (3)	
**Sex**			
	Female	32 (82)	
	Male	7 (18)	
**Physical activity**			
	Sedentary	1 (4)	
	Low active	20 (43)	
	Active	15 (33)	
	Very active	3 (7)	
**Medical conditions**			
	No conditions	36 (91)	
	Hypertension	3 (8)	
	Diabetes	1 (2)	

#### Objective Measurement Results

**Figure 4:**
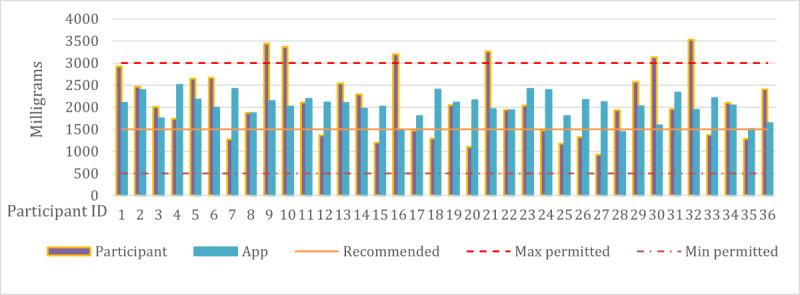
Comparison of average daily sodium content of meals planned by nonhypertension participants and the app.

First, we compared meal nutrition in terms of PV values for user-designed plans and AI-designed plans. The PV for user-designed plans is significantly lower (<0.2). This indicates a lesser alignment with the optimal recommended nutrient values, when compared with the PV of meals planned by the app (0.8). These results demonstrate that the proposed planning system optimized PV values, that is, providing excellent nutrition for participants’ health. The breakdown of PV values for each of the 3 patient groups: diabetes (n=1), hypertension (n=3), and no concerns (n=35) is worth noting. For those with diabetes, the average PV of participants' meal plans was 0.0561, which substantially improved to 0.8125 with the app's meal plans. Similarly, participants with hypertension initially had an average PV of 0.0305, which increased to 0.7895 when using the app’s meal plans. Even those without any health concerns showed an improvement from a PV of 0.0873 to 0.801 using the app. This reveals that due to their health concerns and subsequent dietary constraints, it is more challenging for individuals with health issues to achieve higher PV values. Hence, the meals provided by the app can dramatically improve the nutritional values required by people with health concerns. We analyzed all the nutrients in meals recommended by the app and compared them with those planned by the participants. As an example, we’ve presented the different daily sodium content in the meals of participants’ plan and the app’s plan, as shown in Figure 4. Note that the partisans in the figure do not have any health concerns. The 3 horizontal lines indicate the maximum, recommended, and minimum permitted amounts of sodium consumption per day. For each participant, there are 2 sodium values: the yellow one is sodium from meals planned by the participant themselves, whereas the blue one is the sodium content from meals planned by the app for that participant. We can see that the app’s sodium contents are closer to the recommended amount of sodium consumption. In some cases, the participants’ sodium contents exceeded the maximum limit. This figure highlights the effectiveness of the proposed planning in controlling sodium intake compared with the participant-designed plans.

**Figure 5:**
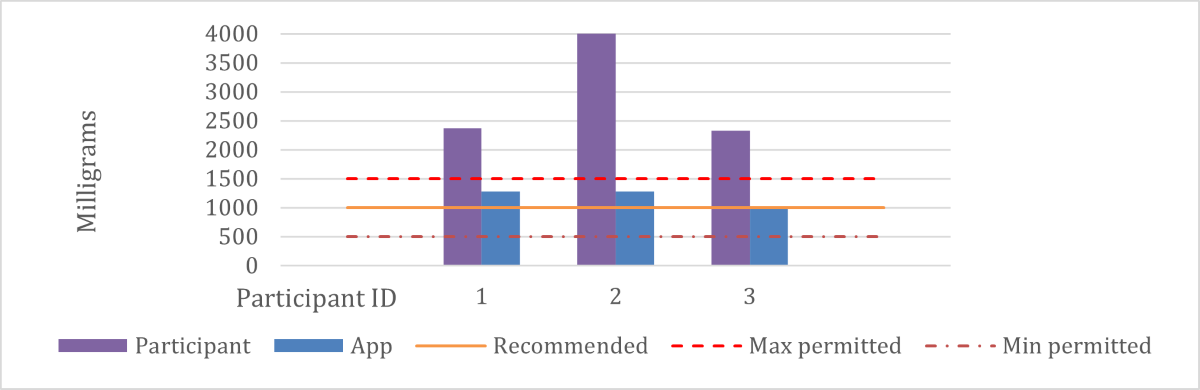
Comparison of the average daily sodium content of meals planned by participants of hypertension and the app.

Figure 5 shows the sodium content of the participants with hypertension. We separated them because of the different sodium limits specified in the health diet guidelines for this group. The results showed that all users with hypertension exceeded the maximum limit in their sodium intake, whereas the app’s plan was closer to the recommended limit.

**Figure 6:**
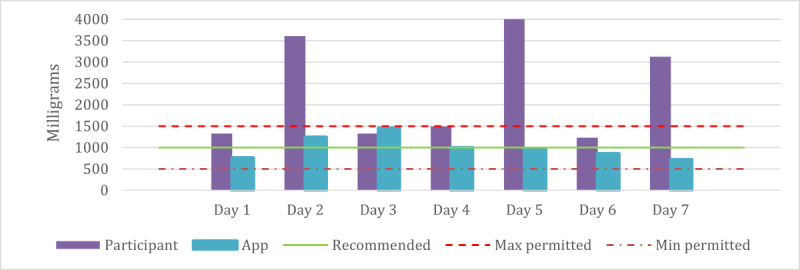
Comparison of the weekly sodium content of meals planned by participants of hypertension and the app.

Figure 6 shows the sodium content of a hypertensive participant over 1 week. It provides a clear illustration of daily sodium intake, allowing a more in-depth analysis of the individual’s diet.

The analysis of the app's adherence to user preferences reveals some significant insights. The preference scores were compared between the participant-designed plans and the app-designed plans, utilizing the TOPSIS score (as shown in equation 11 in Multimedia Appendix 1). The app-designed plans registered higher scores, suggesting that the app was more successful in closely adhering to individuals' desired preferences.

Interestingly, we found that when participants created their plans, they usually focused on 1 or 2 preferences, although they had expressed multiple preferences in their survey. This tendency resulted in a TOPSIS score of 0.4564 for participant-designed plans. On the other hand, the app-designed plans, which took into account a wider range of user preferences, achieved a higher TOPSIS score of 0.5227. This result demonstrates that the app effectively considered people’s preferences in their meal plans, offering a more customized and inclusive meal planning experience.

#### Subjective Measurement Results

To evaluate the participants’ overall satisfaction with the mobile app, we asked them to answer a questionnaire with 3 questions regarding the app’s usefulness, time savings, and consideration of their diet constraints and preferences. All the participants completed the questionnaire after using the app. The results showed that most of the participants were satisfied with the mobile planning system and had a positive view of its features. As our data shows, most of the participants had a positive view of whether the app was helpful for meal planning. Specifically, 13% (5/39) were extremely positive, 54% (21/39) were very positive, and 31% (12/39) were somewhat positive. Only 3% (1/39) of the respondents had a negative view. Our data shows that most participants agreed that the app helped them save time in meal planning. Specifically, 33% (13/39) were extremely positive, 36% (14/39) were very positive, 26% (10/39) were somewhat positive, and only a minor proportion of 5% (2/39) were not positive.

Our findings illustrate that most respondents had a positive view of the app, considering their diet constraints and overall preferences in meal planning. Specifically, 23% (9/39) were extremely positive, 36% (14/39) were very positive, 31% (12/39) were somewhat positive, and 10% (4/39) were not positive.

## Discussion

### Principal Findings

This study proposed a personalized healthy meal planner for people who would like to eat healthily, especially for people with diet-related health concerns and compared the nutritional value of meals planned by users and by an AI-based mobile planning system. The results showed that the AI system optimized the nutrient value, providing better nutrition for the participants. The system was also more successful in addressing health-related nutrition intake (eg, controlling sodium intake) and following participants’ dietary preferences, as shown by higher scores in terms of TOPSIS scores. Participants were satisfied with the mobile planning system, with most having a positive view of the app’s helpfulness in meal planning, saving time, and considering their dietary constraints and preferences.

### Comparison With Prior Work

Existing meal planning systems can be broadly classified into 2 categories: manual and automated systems. Manual meal planning systems typically involve the use of cookbooks, recipes, and nutritional guides to plan and prepare meals. These systems can be time consuming, and the results depend on the user’s knowledge and ability to find appropriate recipes and ingredients.

Automated meal planning systems [37,38] use technology to help with meal planning. These systems can be web-based [6-8,22], mobile app–based [19,20,39], or integrated into smart kitchen devices [40]. Automated meal planning systems typically use algorithms to suggest meals based on the user’s dietary needs, preferences, and restrictions [38,41-43]. They may also consider other factors such as food availability, cost, and time constraints [44,45]. Some popular examples of automated meal planning systems include MyFitnessPal [16], Noom [46], and Mealime [39].

The proposed AI meal planning system integrates multiple techniques, such as semantic reasoning, fuzzy logic, heuristic search, and multicriteria analysis, to create optimized meal plans tailored to the user’s individual needs and preferences. It uses an ontology-based knowledge base, semantic rules, and fuzzy membership functions to handle uncertain and vague nutritional data. The system also incorporates a semantic rule-based filter, a heuristic search method, and an MCDM approach to make meal suggestions. A mobile app prototype was developed and tested with positive results, including well-considered health concerns, optimized nutrition values, respect for restrictions and constraints, consideration of user preferences, and user satisfaction.

Compared with existing meal planning systems, this AI-powered system is unique in its integration of multiple techniques to create personalized meal plans that consider multiple factors, such as health conditions, dietary restrictions, and user preferences. In addition, the use of fuzzy logic and heuristic search sets it apart from other systems that may rely on purely deterministic or rule-based methods [41,47,48].

### Limitations

This study had some limitations that need to be acknowledged. One limitation is that the system does not dynamically adjust meal plans to accommodate changes, such as when a user cannot follow the plan on a specific day. This may affect the user’s adherence to and satisfaction with the system. Another limitation is that the system assumes that the user’s health conditions, preferences, and status remain unchanged throughout the meal planning process. This may not reflect the reality of some users who may experience changes in their health or lifestyle. These limitations suggest that the system needs further development to incorporate dynamic and real-time updates that can adapt to the user’s changing needs and circumstances. In our current version of the app, our primary source of guidelines is the American Diabetes Association Guidelines, as we are targeting the US population. We plan to include a feature that allows users to select their country within the app. By doing so, we can provide personalized meal plans that align with the specific guidelines and recommendations of their respective countries.

Another limitation of this study is related to the user study design and sample. The user study was conducted using a relatively small and homogeneous sample of 39 adults who had different health conditions and dietary preferences. This may limit the generalizability and external validity of the results to other populations or contexts. Moreover, the user study only evaluated the usability and performance of the system for 1 week, which may not capture the long-term effects or outcomes of using the system. Participants’ satisfaction with the mobile planning system should extend beyond the use of the app. Additional features, such as the ability to create menus and generate grocery lists, would enhance the overall user experience and determine the system’s usefulness in practical meal preparation. Further investigations are needed to assess the app’s performance in real-world scenarios and gather more comprehensive feedback from users regarding their meal planning and preparation experiences. These investigations would provide a more comprehensive evaluation of the app’s usefulness, usability, and overall user satisfaction. A more comprehensive and longitudinal user study is required to assess how the system improves users’ health and quality of life over time.

### Conclusions

The proposed AI-powered meal planner provides a novel solution for the challenge of healthy and personalized meal planning. The system uses a combination of semantic reasoning, fuzzy logic, heuristic search, and multicriteria analysis to generate optimized meal plans that are tailored to an individual’s unique dietary needs, preferences, and goals. The use of semantic web technologies to model complex and heterogeneous diet knowledge, semantic logic to enable automatic machine reasoning, fuzzy logic to handle the uncertainty and vagueness of food data, and heuristic search–assisted MCDM to effectively integrate multiple user preferences all contribute to the effectiveness of the system. Through comprehensive evaluation tests, the system was shown to be effective in generating healthy and personalized meal plans, and the mobile app prototype demonstrated its feasibility for practical use. However, it is important to note that the system has some limitations that should be addressed in future studies. Overall, this work represents a significant step forward in the development of AI-powered personalized meal planning systems, providing a valuable tool to help people achieve their health and nutritional goals.

## Appendix

Multimedia Appendix 1 Descriptive explanation of fuzzy membership functions for various nutrients, fuzzy nutrition optimization algorithm, and multiobjective optimization algorithm.

## Abbreviations

AI: artificial intelligence
MCDM: multicriteria decision-making
PV: Prerow Value
TOPSIS: Technique for Order of Preference by Similarity to Ideal Solution

## Data Availability

The data sets analyzed during this study are available from the corresponding author upon reasonable request.
