# Supplementary material for: Personalized Flexible Meal Planning for Individuals With Diet-Related Health Concerns: System Design and Feasibility Validation Study
Source: JMIR Form Res. 2023 Aug 3;7:e46434. doi: 10.2196/46434 (PMC10436119; doi:10.2196/46434)
Supplement: Multimedia Appendix 1 [file formative_v7i1e46434_app1.docx]

## Multimeddia Appendix

### A.1 Fuzzy membership function of different nutrients

Fuzzy logic is a way of representing vague information with no discernible start or end. In contrast, a crisp set is a way of representing clear information with a definite boundary. An object either belongs or does not belong to a crisp set. Most of the current diet rules and guidelines are based on Dietary Reference Intakes (DRI) using crisp sets. DRI provides a range of allowances for nutrition expressed in crisp numbers. For instance, the optimal range of a dietary intake x_a_ can be described in the crisp formulation as follows [27,29]:

Equation 1

$x_{a,min}\leq x_{a}\leq x_{a,max}$

The crisp membership function µ(x_a_) of x_a_ then can be defined as follows:

Equation 2

$\mu\left( x_{a} \right)=\left\{ \begin{aligned} 1, & x_{a,min}\leq x_{a}\leq x_{a,max} \\ 0, &otherwise \end{aligned} \right.$

The crisp membership function is illustrated by the red solid line in Figure S1. However, fuzzy membership function is a mathematical function that assigns a degree of membership to each element of a fuzzy set. The degree of membership ranges from 0 to 1, where 0 means, the element does not belong to the fuzzy set at all, and 1 means the element fully belongs to the fuzzy set. This approach can be used to model the desirability or optimality of nutrient intake based on dietary recommendations.

To model the membership function µ(x_a_) of a nutrient a, we used a curve-fitting approach. We define key points (extreme points and optimal points) according to rules and guidelines. For ideal points, membership is one, and membership of safe points can be 0.9. In addition, deprived or toxic points have membership zero. We determine the fit curve based on these points. For example, five points are needed to construct the model in Figure 13, including (a) zero intakes, (b) safe minimum limit, (c) optimal intake, (d) safe upper limit, and (e) the toxic extreme intake.

Figure S1-Membership function

|   *Figure S2-membership function graph of protein* |   *Figure S3-membership function graph of fat* |
| --- | --- |
|   *Figure S4-membership function graph of fiber* |   *Figure S5-membership function graph of sugar* |
|   *Figure S6-membership function graph of sodium for a user not suffering from hypertension* |   *Figure S7-membership function graph of sodium for users suffering from hypertension* |
|   *Figure S8-membership function graph of carbohydrates for a user not suffering from diabetes* |  |

Figures S2-8 show the fuzzy membership function of some different nutrients we modeled. For example, Figure S2 models the protein membership. Based on the healthy-eating guidelines, a person’s protein consumption should account for 20% to 30% of his/her calorie intake and no less than 10% or over 50%. For a person with an approximate 1200 k calorie energy requirement each day, 10%, 20%, 30%, and 50% calories correspond to 52.5, 105, 157.5, and 262.5 grams of protein respectively. Using these four points we can model the protein membership as shown in Figure S2. Similarly, we can model the membership of fat, fiber, sugar, sodium, and carbohydrates (Figures S3-8). For people with hypertension and people without hypertension, the sodium intake recommendations are different. Therefore, the sodium membership in Figure S6 and Figure S7 are different. Figure S6 shows the sodium membership model for people without hypertension, while Figure S7 is for hypertension. A major difference is in the toxic point, which is 5000mg for a normal user, and 2300mg for someone with hypertension.

### A.2 Fuzzy nutrition optimization algorithm

Fuzzy nutrition optimization algorithm is an algorithm that uses fuzzy logic and heuristic search to find the best combination of meals for a day (breakfast, lunch, and dinner) that have optimal nutrition values based on the user's health concerns. The algorithm uses a fuzzy membership function to evaluate the desirability of nutrient intake and a Prerow Value (PV) to measure the closeness of a meal's nutrients to the optimal recommended value. PV is the product of the minimal membership value and the harmonic mean to the fuzzy sets of the rest of the observed nutrients, as defined in Equation 3. PV is graded between 0 and 1, and nutrition with the lowest value has the most influence on the result.

Equation 3

$PV=min\left[ \mu\left( x_{i} \right) \right]\cdot\left( n-1 \right)\cdot\left( \sum_{i\neq i_{min}} \frac{1}{\mu\left( x_{i} \right)} \right)^{-1}$

Based on Wirsam’s research, preferred PV values are greater than 0.7 and optimal PV values are greater than 0.9 [28]. The algorithm also uses a population of distinct meals as candidates for an optimal daily meal and iteratively improves each meal by replacing the meal that has the lowest PV with a better meal that has a better amount of nutrition. The algorithm stops when all the meals in the population have a PV greater than 0.9, which means they are optimal.

The detailed algorithm is listed in the following steps:

| *1. Define the fuzzy membership function for all nutrients based on dietary recommendations and curve-fitting approach (see Appendix A.2 for details).*  *2. Generate a random initial array of n (n>M) daily packs of meals. Each pack contains three meals: breakfast, lunch, and dinner.*  *3. Sort the array based on PV values and put M best ones to the Global-Best list.*  *4. While (true) {*  *- For Element i=1 to n {*  *- If PV(Elementi) < 1 {*  *- Find nutrient j (with nutrient amount T) that has the lowest membership value in Elementi (i.e., µ(T) is the minimum in element i)*  *- Find meal k of Elementi (k ∈ {breakfast, lunch, diner} with nutrient amount S) dominating the loss of j’s membership*  *- Find a meal, q, in the meal set, whose nutrient j’s amount is Y, so that µ(T-S +Y)=1*  *- Replace meal k with q in element i.*  *}*  *- Calculate the new PV value, PV(Elementi)*  *- If PV(Elementi)> Global-Best (M) {*  *- Global-Best <-Elementi*  *- Sort Global-Best*  *}*  *- If Global-Best (M) >0.9*  *- Break*  *}*  *}* |
| --- |

The following example demonstrates how the algorithm works. In the beginning, we have three meal options for breakfast, lunch, and dinner listed in Table S1. The fitness value of the meals of the day is PV=0.15. The detailed nutrition distribution is shown in Table S1. Sugar has the lowest membership value and lunch has the highest amount of sugar.

*Table S1- Calculating PV for suggested meals in iteration i_th_*

| Meal Nutrition | | Carbohydrate | Sodium | Sugar | Fiber | Protein | Fat | $PV=0.2\times\left( 6-1 \right)\times\left( \frac{1}{0.81}+\frac{1}{0.55}+1+1+1 \right)^{-1}=0.15$ |
| --- | --- | --- | --- | --- | --- | --- | --- | --- |
| Breakfast | Breakfast Granola | 75.7 | 939.7 | 29.9 | 11.6 | 17.6 | 28 |  |
| Lunch | Lemony Glazed Carrots | 68.6 | 168.9 | 59.4 | 4.6 | 1.2 | 0.3 |  |
| Diner | Easy Awesome Tuna Casserole | 86 | 777 | 3.7 | 4.1 | 31.1 | 21.9 |  |
| Total Nutrition in a day | | 178.4 | 1885.6 | 93 | 20.3 | 49.9 | 50.2 |  |
| Nutrition membership value in a day | | 1 | 1 | 0.2 | 0.81 | 0.55 | 1 |  |

Therefore, in the next iteration, lunch will be replaced with a meal with a lower amount of sugar. The meal of the day after the lunch replacement is shown in Table S2: With the replacement, the new fitness score is improved to PV=0.86.

*Table S2-Calculating PV for suggested meals in iteration i_th_+1 after lunch replacement*

| Meal Nutrition | | Carbohydrate | Sodium | Sugar | Fiber | Protein | Fat | $PV=0.868\times\left( 6-1 \right)\times\left( 1+1+1+1+1 \right)^{-1}=0.86$ |
| --- | --- | --- | --- | --- | --- | --- | --- | --- |
| Breakfast | Breakfast Granola | 75.7 | 939.7 | 29.9 | 11.6 | 17.6 | 28 |  |
| Lunch | Salmon and Basil Quinoa Salad | 52 | 53.4 | 0 | 6 | 35.1 | 11.5 |  |
| Diner | Easy Awesome Tuna Casserole | 86 | 777 | 3.7 | 4.1 | 31.1 | 21.9 |  |
| Total Nutrition in a day | | 213.7 | 1770 | 33.6 | 21.7 | 83.8 | 61.4 |  |
| Nutrition membership value in a day | | 1 | 1 | 1 | 0.868 | 1 | 1 |  |

If the new PV is better than the worst PV in the Global-Best list, the worst meal will be replaced with the new meal and the Global-Best list will be sorted again. After several iterations, all members in the global-best list have a PV greater than 0.7, which is considered acceptable. It means we have a list of candidate daily meal options with optimal nutrition values based on the user’s health concerns.

### A.3 Multi-objective optimization algorithm

Multi-objective optimization algorithm is an algorithm that uses the combined Technique for Order of Preference by Similarity to Ideal Solution (TOPSIS) and Analytic Hierarchy Process (AHP) methods to find the best meals based on multiple criteria, such as nutrition, taste, cost, and convenience. The algorithm uses a multi-criteria decision-making (MCDM) approach to consider the user's preferences and make an informed decision about which meal is the best option. The algorithm mainly includes defining criteria for choosing the best meal, determining the weighting of each criterion based on the user's preferences using AHP, normalizing the criteria values, calculating the weighted normalization of each meal, determining the positive and negative ideal solutions, calculating the Euclidean distances, determining the relative closeness of each meal to the positive ideal solution, and finally sorting the meals based on the relative closeness values to find the best meal.

The detailed algorithm is listed in the following steps:

1. Define the criteria for choosing the best meals, such as nutrition, taste, cost, and convenience.
2. Determine the weighting of each criterion based on the user's preferences using the AHP method: creating a pairwise comparison matrix N×N using the weighting score and establishing a performance decision matrix $A_{ij}=\left( a_{ij} \right)_{m\times n}$ consisting of $m$ meals and $n$ different preferences:

Equation 4

$A_{ij}=\left[ \begin{matrix} a_{11} & \cdots& a_{1n} \\ \vdots& \ddots& \vdots\\ a_{m1} & \cdots& a_{mn} \end{matrix} \right]$

1. Normalize the criteria values to make sure that each criterion has the same impact on the final decision. Normalize the $A_{ij}$ matrix to the matrix $R=\left( r_{ij} \right)_{m\times n}$

Equation 5

$r_{ij}=\frac{x_{ij}}{\sqrt{\sum_{k=1}^{m} x_{kj}^{2}}}, i=1,2,\cdots,m j=1,2,\cdots,n$

1. Calculate the weighted normalization of each meal based on the criteria values and the weights determined in step 2.

Equation 6

$t_{ij}=r_{ij}\cdot w_{j} , i=1,2,\cdots,m j=1,2,\cdots,n$

Where $w_{j}={W_{j}}/{\sum_{k=1}^{n} W_{k}}$, $j=1,2,\cdots,n$ , so that $\sum_{i=1}^{n} w_{i}=1$ and $W_{j}$ is the original weight given to the indicator $v_{j}$, $j=1,2,\cdots,n$.

1. Calculate the best (positive ideal) solution and the worst (negative ideal) solution based on the weighted normalization values.

Equation 7

$A_{w}=\left\{ \left\langle max\left( t_{ij} | i=1,2,\cdots,m \right) | j\in J_{-} \right\rangle,\left\langle min\left( t_{ij} | i=1,2,\cdots,m \right) | j\in J_{+} \right\rangle\right\}\equiv\left\{ t_{wj} | j=1,2,\cdots,n \right\}$,

Equation 8

$$A_{b}=\left\{ \left\langle min\left( t_{ij} | i=1,2,\cdots,m \right) | j\in J_{-} \right\rangle,\left\langle max\left( t_{ij} | i=1,2,\cdots,m \right) | j\in J_{+} \right\rangle\right\}\equiv\left\{ t_{bj} | j=1,2,\cdots,n \right\}$$

where,

$J_{+}=\left\{ j=1,2,\cdots,n | j \right\}$ associated with the criteria having a positive impact, and

$J_{-}=\left\{ j=1,2,\cdots,n | j \right\}$ associated with the criteria having a negative impact.

1. Calculate the Euclidean distance between each meal and the best solution and the worst solution.

The distance between the target alternative $i$ and the worst condition $A_{w}$

Equation 9

$d_{iw}=\sqrt{\sum_{j=1}^{n} \left( t_{ij}-t_{wj} \right)^{2}}, i=1,2,\cdots,m$

And the distance between the alternative $i$ and the best condition $A_{b}$

Equation 10

$d_{ib}=\sqrt{\sum_{j=1}^{n} \left( t_{ij}-t_{bj} \right)^{2}}, i=1,2,\cdots,m$

Where $d_{iw}$ and $d_{ib}$ are Euclidean distance from the target alternative $i$ to the worst and best conditions, respectively.

1. Calculate the meals similarity to the worst alternative (TOPSIS score), the larger, the better.

Equation 11

$$S_{i}=\frac{d_{iw}}{d_{iw}+d_{ib}}, i=1,2,\cdots,m$$

1. Sort the meals based on the TOPSIS score. The meal with the highest TOPSIS score is the best option according to the user's preferences.
